# Dimensional and positional temporomandibular joint osseous characteristics in normodivergent facial patterns with and without temporomandibular disorders

**DOI:** 10.1007/s00784-023-05120-0

**Published:** 2023-06-26

**Authors:** Maged S. Alhammadi, Abeer A. Almashraqi, Ahmed A. Thawaba, Mona M. Salah Fayed, Amira A. Aboalnaga

**Affiliations:** 1 Department of Preventive Dental Sciences, College of Dentistry, Jazan University, Jazan, Saudi Arabia; 2 Department of Pre-Clinical Oral Health Sciences, College of Dental Medicine, QU Health, Qatar University, Doha, Qatar; 3 Orthodontic Department, Faculty of Dentistry, Mansoura University, Mansoura, Egypt; 4 Department of Orthodontics, Faculty of Dentistry, Cairo University and Vice Dean for Postgraduate Studies and Research, Faculty of Dentistry, MSA University, Cairo, Egypt; 5 Department of Orthodontics, Faculty of Dentistry, Cairo University, Cairo, Egypt

**Keywords:** Cone beam computed tomography, Joint spaces, Mandibular condyle, Normodivergent facial pattern, Temporomandibular joint disorders

## Abstract

**Objective:**

This study evaluated the dimensional and positional osseous temporomandibular joint features in normodivergent facial patterns with and without temporomandibular disorders.

**Methods:**

A total of 165 adult patients were divided into two groups: group 1 (*n* = 79 patients; 158 joints): temporomandibular disorders patients and group 2 (*n* = 86 patients; 172 joints): non-temporomandibular disorders patients. Three-dimensional positional and dimensional temporomandibular joint characteristics, including glenoid fossa, mandibular condyles, and joint spaces, were assessed by cone beam computed tomography.

**Results:**

The glenoid fossa positions in the three orthogonal planes and height showed statistical significance between the two studied groups. The temporomandibular disorders patients showed higher horizontal and vertical condyle inclinations while anteroposterior inclination was less, and the condyle was positioned more superior, anterior, and lateral in the glenoid fossa. The condyle width and length showed no significance between the two groups, while condyle height was smaller in temporomandibular disorders patients. Anterior and medial joint spaces increased while the superior and posterior joint spaces reduced in temporomandibular disorders patients.

**Conclusion:**

There were significant differences between the patients with and without temporomandibular joint disorders in terms of mandibular fossa positions and height as well as condylar positions and inclinations in horizontal and vertical planes together with reduced condylar height and reduced posterior and superior joint spaces in the temporomandibular disorders patients.

**Clinical relevance:**

The temporomandibular disorder is a multifactorial disorder in which one of these factors is the dimensional and positional characteristics of the temporomandibular joints; including or excluding this factor requires a comprehensive three-dimensional investigation of patients with TMD compared to the normal group under the condition that the facial pattern is average as a confounding factor.

**Supplementary information:**

The online version contains supplementary material available at 10.1007/s00784-023-05120-0.

## Introduction

The temporomandibular joint (TMJ) is a complex, delicate, and extensively utilized joint by humans. It articulates the mandible with the temporal bone of the human skull to regulate its movements. It has two condyles at both ends of the mandible and functions simultaneously [1]. TMJ has a unique mechanism in which there is no contact between the articular surfaces of the bones with each other. However, they are separated by a disc that serves as a cushion for stress absorption and permits the easy movement of condyles when the oral cavity is opened and closed. This disc splits TMJ into two synovial cavities, with synovial membranes lining them. The articulating surfaces of bones are covered with fibrocartilage, not hyaline cartilage [2].

Temporomandibular disorder (TMD) is a generic term comprising a heterogeneous group of complex diseases of variable and usually multifactorial etiologies affecting the masticatory musculature of the head and neck, osseous structures of the human mandible and TMJ, and soft tissue structures of TMJ such as the disc and its attached ligaments. Injuries involving the mandible, TMJ, or head and neck muscles could result in TMD. Other potential etiologies are teeth grinding or clenching, which increases the pressure on TMJ; disc dislocation; osteoarthritis or rheumatoid arthritis involving TMJ; psychosocial stress and its associated tightening of muscles of the face and jaw; and aging effects [3].

There is high inter-individual variability in TMD-related signs and symptoms; however, they are divided into six major groups: (1) pain dysfunction syndrome, non-dental pain involving the orofacial region, which is the most common TMJ disorder, and these individuals often complain of pain on mastication; (2) joint noise: clicking, crepitation, and grinding; (3) TMJ locking: incapability of complete closing or opening; (4) tender muscles in patient’s face, neck, and a shoulder; (5) ear symptoms: otalgia, tinnitus; and (6) psychosocial complaints [4, 5].

The incidence of TMD had been shown to be higher in the general population (20–75%) compared with an incidence of 2–4% in those who presented to receive therapy [6]. TMD is often presented in the second to the fourth decade, and there are no sex differences in symptoms (1:1). However, there are significantly more female patients than male patients seeking therapy, with a ratio of 7:1 [7].

The recent high-level evidence showed that TMD prevalence in patients seeking orthodontic treatment ranged from 21.1 to 73.3%; the percentage of males and females presenting with TMD varied from 10.6 to 68.1% and 21.2 to 72.4%, respectively [8]. Another recent systematic review and meta-analysis concluded that the prevalence overall meta-analyses for adults/elderly are as follows: TMD (31.1%), disc displacements (19.1%), and degenerative joint disease (9.8%). Furthermore, for children/adolescents, they are as follows: TMD (11.3%), disc displacements (8.3%), and degenerative joint disease (0.4%) [9]. The most recent systematic review with meta-analysis evaluated the prevalence of temporomandibular disorders in children and adolescents using Diagnostic Criteria for Temporomandibular Disorders (DC/TMDs) showed that among 1093 female, 489 (44.7%) presented TMD, while 247/821 male (30%) experienced TMD and overall TMD prevalence in children and adolescence varies between 20 and 60%. Females had a higher prevalence of TMDs compared to males [10].

Many radiographic techniques have been utilized for the assessment of the morphological and positional features of soft and hard tissue components of TMJ using conventional 2D imaging, multidetector computed tomography (MDCT), magnetic resonance imaging (MRI), and computed tomography (CT) [11, 12]. However, the most common limitation of using conventional 2D radiography is the superimposition of neighboring structures of TMJ [13]. Recently, cone beam computed tomography (CBCT) has been utilized to produce high-resolution images with little distortion. It is more rapid with a smaller irradiation dose than CT. The measurement of the length and volume in multiple planes can be obtained by a three-dimensional (3D) CBCT scan, giving a correct diagnosis and good predictability of therapeutic outcomes [14].

The temporomandibular disorder is a multifactorial disorder in which one of these factors are the dimensional and positional characteristics of the temporomandibular joints; including or excluding this factor requires a comprehensive three-dimensional investigation of patients with TMD compared to the normal group under the condition that the facial pattern is normal as a confounding factor. To our knowledge, there was no comparative study conducted that evaluated comprehensively the positional and morphologic structures of TMJ in adult patients with and without TMD. Thus, the current study was designed for 3D evaluation of the dimensional and positional osseous TMJ parameters in normodivergent facial patterns with and without TMD.

## Materials and methods

### Study design

The current cross-sectional study obtained its approval from the research ethics committee of the Faculty of Dentistry, Cairo University, Egypt (No. 2152012). The procedures were carried out following the relevant laws and regulations. Every patient was informed about the goal and methods of study, and then they provided written consent.

### Sample size and selection

The sample size was calculated based on *α* value of 0.05 and a power of 95% according to Al-Rawi et al. [15] study in which the mediolateral (ML) measurements of the condyle were 18.98 ± 2.55 and 15.81 ± 3.05 mm in the studied groups. The sample size was calculated to be at least 22 joints in each studied category. However, such a number was increased to at least 30 joints in each group.

Patients were considered desirable based on the following general inclusion criteria: (1) age 18–30 years; and (2) patients have all permanent teeth erupted except for the third molars, while the specific inclusion criteria for the normal group were patients without a history of TMD and/or jaw muscles, and painful or limited movement of the mandible; and the specific inclusion criteria for TMD group were patients with a history of TMD including either disc displacement with or without reduction [16]. The exclusion criteria were: (1) patients with a history of growth abnormalities, condylar degenerative illnesses (e.g., erosion, subchondral cysts, and condylar hyperplasia) [17], polyarthritis, acute trauma, or tumors of TMJ; (2) patients with a history of medications which can influence the TMDs; and (3) patients with a history of orthodontic therapy or had orthognathic or TMJ surgeries. One hundred sixty-five patients (330 joints) who met the previously mentioned inclusion and exclusion criteria were selected out of 1063 individuals who were examined at the outpatient clinic of the Orthodontic Department, Faculty of Dentistry, Cairo University, Egypt.

### Clinical examination

Two operators (A.A. and M.A.) carried out the clinical examination under the direct supervision of an experienced TMD specialist (M.F.). Additionally, before the start of the research, the measurements of a pilot sample of thirty subjects, which were recorded by the three operators, were calibrated with the specialist’s measurements, and inter-observer reliability (A.A.) was statistically determined. Customized history and examination chart were utilized following the DC/TMDs [16]. Clinical evaluation of the enrolled patients included: (1) TMJ palpation; (2) masticatory muscles evaluation and palpation; (3) mandibular movements evaluation; and (4) TMJ sounds assessment.

The total sample was divided into two groups: group 1: TMD group (79 patients; 158 joints) and group 2: non-TMD group (86 patients; 172 joints). Patients were examined using the examination chart following DC/TMD. The CBCT was used to assess the 3D positional and dimensional characteristics of TMJ, which included the glenoid fossa, mandibular condyles, and the TMJ spaces.

### CBCT analysis

Three-dimensional images were acquired by I-CAT CBCT system (Imaging Sciences International, Hatfield, USA) at the Faculty of Dentistry, Cairo University, Egypt. The machine set with the following exposure parameters: 18.54 mAs and 120 kV, and images underwent capture for 8.9 s with a 0.30-mm voxel size, 2 mm slice thickness, and large field of view (17 cm^2^). CBCT images were captured in Frankfort horizontal (FH) plane reoriented parallel to the floor aided by crossing laser guide, and teeth were occluded in centric occlusion (CO). Then, the midsagittal reference plane was automatically set. This plane was perpendicular to FH plane and passed through Nasion. During the scanning process, patients were informed to avoid swallowing or movement during scanning process.

**Fig. 1:**
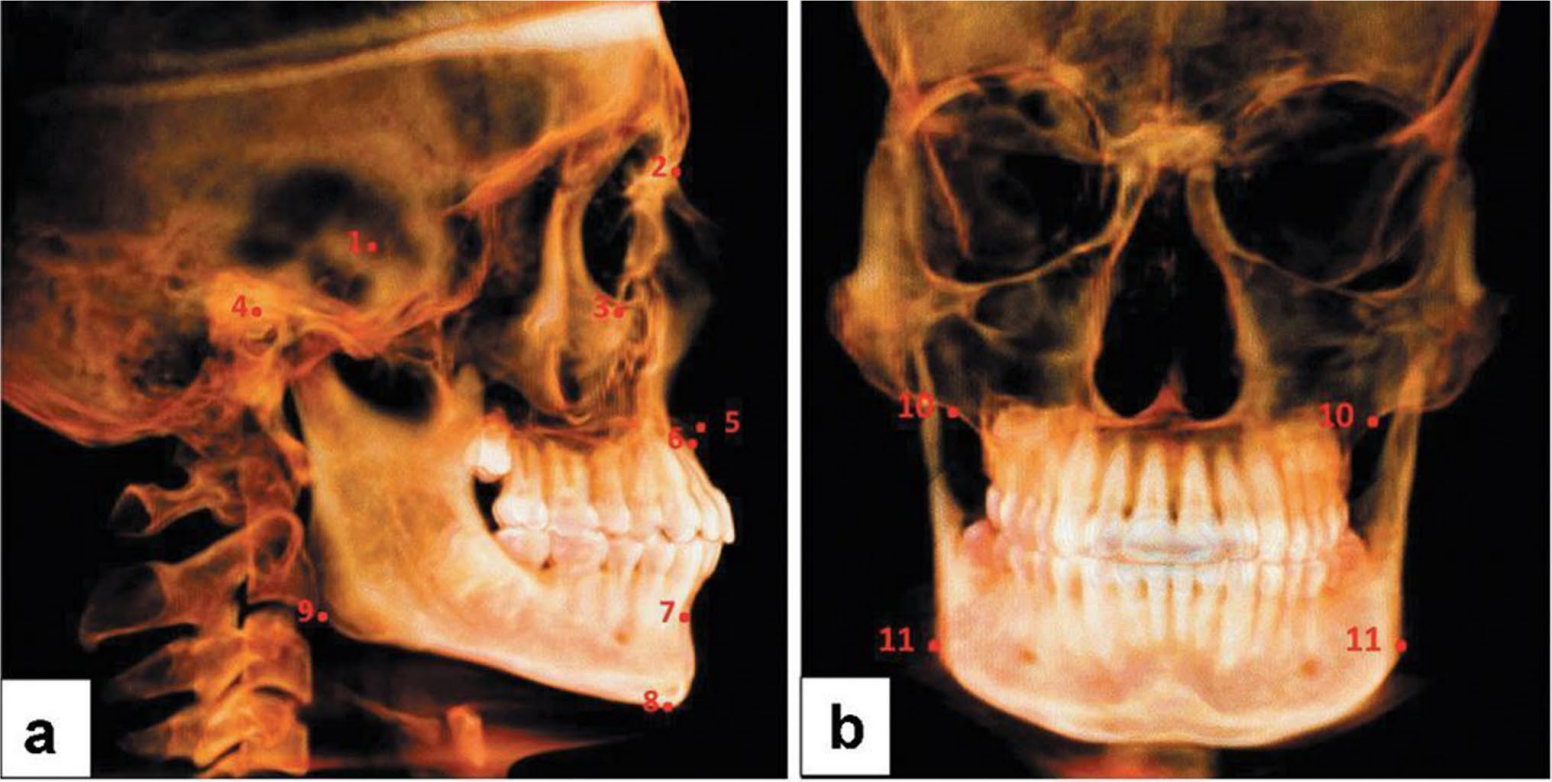
Three-dimensional skeletal landmarks: **a** anteroposterior landmarks and **b** mediolateral landmarks

**Fig. 2:**
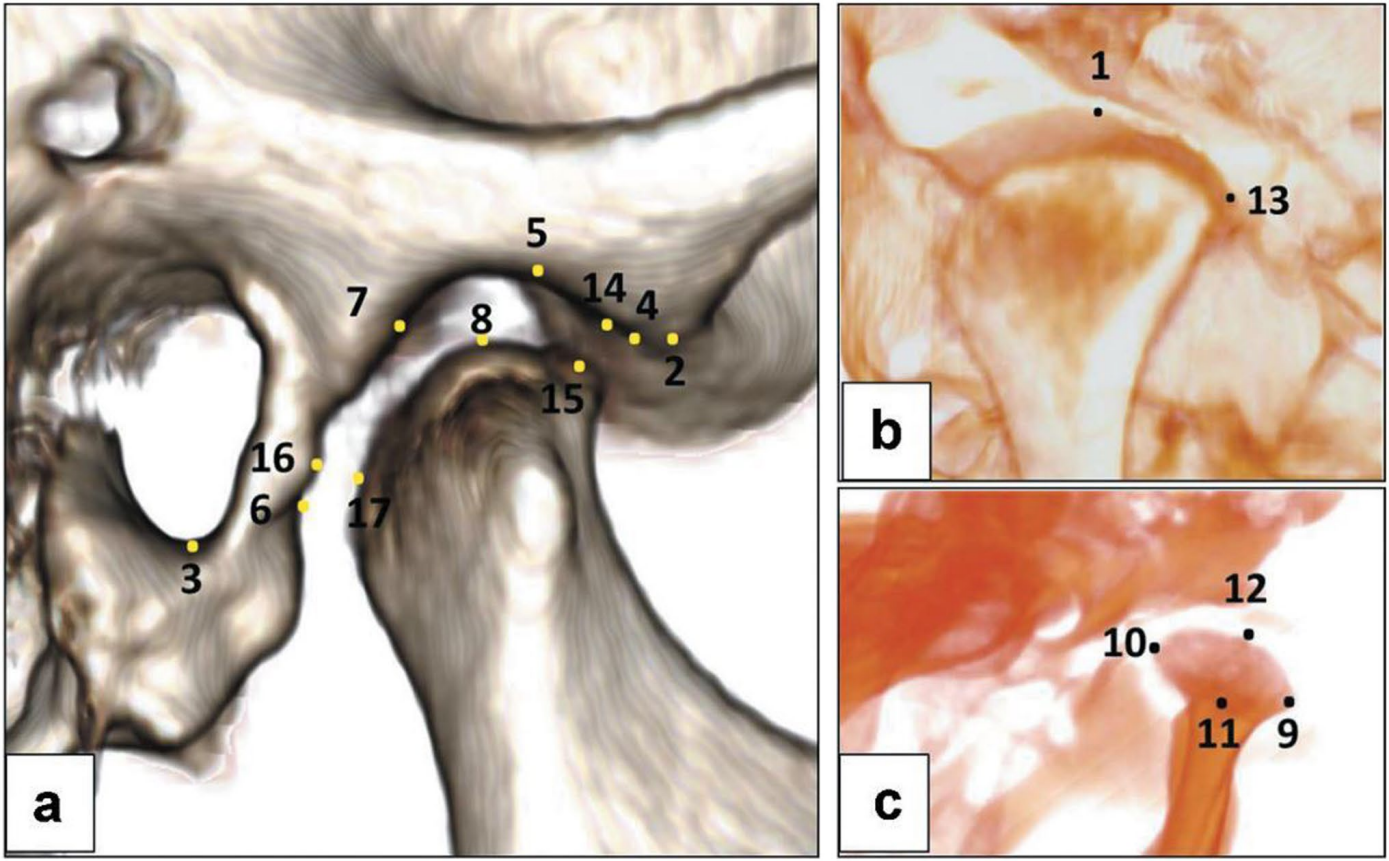
Three-dimensional temporomandibular joint landmarks: **a** sagittal view, **b** coronal view, and **c** axial view

CBCT images were acquired based on Digital Imaging and Communications in Medicine (DICOM) files and then exported to Invivo Anatomage 5.01 (Anatomage, San Jose, USA) for 3D analysis. The landmarks of craniofacial structures and TMJ were recognized in a 3D view and underwent adjustment in the three orthogonal planes (Table 1 and supplementary material 1) by slice locator option (Figs. 1 and 2). The standardized innovative 3D imaging of all linear and angular measurements of craniofacial images described by Alhammadi et al. [18–20] was used in this study and described in Table 2 and supplementary material 2. Positional and dimensional mandibular fossa and condylar osseous parameters relative to skull base reference were evaluated. The analysis included TMJ joint spaces, anterior (AJS), superior (SJS), posterior (PJS), and medial joint space (MJS). The anteroposterior and vertical condylar position inside the joint was calculated based on the formula developed by Pullinger and Hollender [21].

**Table 1:** Definitions of skeletal and temporomandibular three-dimensional landmarks used in the study

No	Landmark	Definition
Skeletal landmarks (Fig. 1)		
1	S	The center point of the pituitary fossa in the middle cranial fossa in sagittal and axial views
2	N	The most anterior and midpoint of the fronto-nasal suture
3	Or	The most inferior and middle point of each infra-orbital rim
4	Po	The most outer and superior bony points of the external acoustic meatus
5	ANS	The most anterior midpoint of the anterior nasal spine of the maxilla
6	A point	The deepest midpoint of the maxillary anterior surface
7	B point	The deepest midpoint of the mandibular anterior surface
8	Me	The most inferior midpoint of the chin on the outline of the mandibular symphysis
9	Go	The right and the left midpoint on the angles of the mandible, halfway between the corpus and ramus
Temporomandibular landmarks (Fig. 2)		
1	MF	The most superior and midpoint of the hard tissue right or left mandibular fossa region
2	AT	The most inferior point of the right or left articular tubercle
3	IM	The most inferior point of the right or left internal auditory meatus
4	AFPi	The most anterior and inferior point in the right or left anterior wall of the mandibular fossa
5	AFPs	The most superior point in the right or left anterior wall of the mandibular fossa
6	PFPi	The most posterior and inferior point in the right or left anterior wall of the mandibular fossa
7	PFPs	The most superior point in the right or left posterior wall of the mandibular fossa
8	SCP	The most right or left superior point of the condylar head
9	LCP	The most right or left lateral point of the condylar head
10	MCP	The most right or left medial point of the condylar head
11	ACP	The most right or left anterior point of the condylar head
12	PCP	The most right or left posterior point of the condylar head
13	MJSF	The most right or left lateral point of the medial wall of mandibular fossa
14	AJSF	The most posterior point of the right or left anterior wall of the mandibular fossa opposed to the shortest anterior condylar-fossa distance
15	AJSC	The most anterior point of the right or left condyle opposed to the shortest anterior condylar-fossa distance
16	PJSF	The most anterior point of the right or left posterior wall of the mandibular fossa opposed to the shortest posterior condylar-fossa distance
17	PJSC	The most posterior point of the right or left condyle opposed to the shortest posterior condylar-fossa distance

**Table 2:** Reference planes, skeletal, and temporomandibular joint measurements used in the study

3D skeletal reference planes		
HP	Horizontal plane	Constructed by three-point right orbital with two sides portion
MSP	Midsagittal plane	Constructed by three-point N, S, and ANS
VP	Vertical plane	Constructed Sella point and perpendicular to the sagittal and horizontal plane
TM	Tuberculo-metal line	The line between AT and IM
3D skeletal measurements		
ANB	Skeletal anteroposterior jaw relation	The angle between A point, N point, and B point
MP/SN	Skeletal vertical jaw relation	The angle between Sella-Nasion (SN) and Go-Me
3D skeletal temporomandibular joint measurements		
MFPVP	Mandibular fossa vertical position	The perpendicular distance between MF and HP
MFPAP	Mandibular fossa anteroposterior position	The perpendicular distance between MF and VP
MFPML	Mandibular fossa mediolateral position	The perpendicular distance between MF and MSP
GFH	Mandibular fossa height	The perpendicular distance between MF and TM line
GFW	Mandibular fossa width	The horizontal distance between AFPi and PFPi
AFLHP	Mandibular fossa anterior wall inclination	The angle between AFPi, AFPs, and HP
PFLHP	Mandibular fossa posterior wall inclination	The angle between PFPi, PFPs, and HP
HCI	Mandibular condyle horizontal inclination	The angle of ACP-PCP line with HP
VCI	Mandibular condyle vertical inclination	The angle of MCP-LCP line with VP plane
APCI	Mandibular condyle anteroposterior inclination	The angle of MCP-LCP line with MSP
VCP	Mandibular condyle vertical position	The perpendicular distance between SCP and HP
APCP	Mandibular condyle anteroposterior position	The perpendicular distance between ACP and VP
MLCP	Mandibular condyle mediolateral position	The perpendicular distance between MCP and MSP
CL	Condylar length	The distance between MCP and LCP
CW	Condylar width	The condyle distance between CAP and PCP
CH	Condylar height	The perpendicular distance between SCP and a line passing through the constricted condylar neck points
AJS	Anterior joint space	The closest distance between AJSC-AJSF
PJS	Posterior joint space	The closest distance between PJSC-PJSF
SJS	Superior joint space	The closest distance between SCP-MFS
MJS	Medial joint space	The closest distance between MCP-MJSF
VCJP	Vertical condylar joint position	The difference between condyle height to TM line and condyle height to the condyle neckline
APCJP	AP condylar joint position	The anteroposterior position of condyle according to Pullinger and Hollander equation [21]

To evaluate the significance of any measurement errors, 30 cases underwent random selection and were measured twice, 2 weeks apart, by the same operator (M.A.) and once by another operator (A.A.) to assess intra- and inter-observer reliability.

### Statistical analysis

The data were analyzed by IBM-SPSS program (IBM Corp. Released 2019, IBM SPSS Statistics for Windows V 26.0. Armonk, NY). The reliability and reproducibility of measurements were analyzed utilizing the intra-class correlation coefficient (ICC), and the quantitative data were first tested for normality by Shapiro–Wilk’s test and were considered normally distributed if *P* > 0.050 and all data were represented as means ± standard deviations (SDs). An independent *t*-test was utilized to compare normally distributed quantitative data between both groups. The significance of a result was set at *P*-value less than 0.05.

## Results

Regarding the baseline anteroposterior (AP) and vertical (V) skeletal measurements, no significant differences existed between both groups indicating comparable skeletal class and normodivergent facial patterns. The mean point A-Nasion-point B (ANB) angles in TMD and non-TMD groups were 3.9 ± 2.58 and 3.3 ± 2.97°, respectively, and the mean mandibular plane to Sella-Nasion (MP/SN) angles were 34.68 ± 3.39 and 34.97 ± 3.41° in TMD and non-TMD groups, respectively (Table 3).

**Table 3:** Comparative statistical analysis of the baseline anteroposterior and vertical skeletal measurements between the TMD and non-TMD groups

Craniofacial measurements		Group		*P*-value
		TMD group *N* = 79	Non-TMD group *N* = 86	
		Mean ± SD	Mean ± SD	
Anteroposterior	ANB	3.9 (2.58)	3.3 (2.76)	**0.99**
Vertical	MP/SN	34.68 (3.39)	34.97 (3.41)	**0.59**

For mandibular fossa measurements (Table 4), the mandibular fossa positions, the AP, V, and ML, revealed significant differences among both groups. The mandibular fossa parameters showed no statistical significant differences regarding glenoid fossa width (GFW), mandibular fossa anterior wall inclination (AFLHP), and mandibular fossa posterior wall inclination (PFLHP). In contrast, glenoid fossa height (GFH) showed statistical significance between both groups.

**Table 4:** Comparative statistical analysis of the mandibular fossa measurements between the TMD and non-TMD groups

Mandibular fossa measurements		Group				*P*-value
		TMD group		Non-TMD group		
		Mean	SD	Mean	SD	
Mandibular fossa position	MFPVP	1.62	0.97	0.76	0.99	**0.000**
	MFPAP	8.75	2.45	10.20	2.92	**0.001**
	MFPML	47.06	2.65	46.1	2.56	**0.019**
Mandibular fossa parameters	GFH	8.66	1.11	8.28	0.91	**0.019**
	GFW	17.50	1.69	16.99	2.02	**0.083**
	AFLHP	54.14	10.24	52.00	11.59	**0.212**
	PFLHP	48.71	10.86	47.33	10.17	**0.402**

The mean mandibular condyle inclination (Table 5) in horizontal (HCI) and vertical (VCI) planes demonstrated highly significant differences between both groups with *P* < 0.000, while anteroposterior mandibular condyle inclination (APCI) showed no significance in which horizontal and vertical condyle inclinations relative to horizontal (HP) and vertical planes (VP) were higher in the TMD group (6.32 ± 3.60 and 79.39 ± 6.20°, respectively) than the non-TMD group (4.41 ± 2.48 and 75.08 ± 6.60°, respectively). However, anteroposterior condyle inclination relative to the midsagittal plane (MSP) was lesser in the TMD group (73.76 ± 6.50°) than in the non-TMD group (75.50 ± 5.29°).

**Table 5:** Comparative statistical analysis of the mandibular condyle measurements between the TMD and non-TMD groups

TMJ condyle measurement		Group				*P*-value
		TMD group		Non-MD group		
		Mean	SD	Mean	SD	
Mandibular condyle inclination	HCI	6.32	3.60	4.41	2.48	**0.000**
	VCI	79.39	6.20	75.08	6.60	**0.000**
	APCI	73.76	6.50	75.50	5.29	**0.063**
Mandibular condyle position	VCP	1.80	1.24	3.42	1.57	**0.000**
	APCP	4.51	2.37	5.73	2.41	**0.001**
	MLCP	41.48	4.04	39.18	2.36	**0.000**
Mandibular condyle parameters	CL	17.95	2.35	18.52	1.68	**0.075**
	CW	6.85	1.15	7.21	1.28	**0.063**
	CH	8.92	1.41	9.40	1.48	**0.033**
Intra-joint condylar position	APJCP	− 7.09	20.84	11.88	17.75	**0.000**
	VJCP	3.74	0.93	3.28	1.05	**0.003**

As regards condylar positions (Table 5), relative to the basal reference, our results revealed highly significant differences between both groups in all planes; the TMD group showed more superior (VCP), posterior (APCP), and lateral (MLCP) condyle positions (1.80 ± 1.24, 4.51 ± 2.37, and 41.48 ± 4.04 mm, respectively) as compared to the non-TMD group (3.42 ± 1.57, 5.73 ± 2.41, and 39.18 ± 2.36 mm, respectively).

For the mandibular condyle parameters (Table 5), the results showed no statistical significant differences in the condyle length and width in both groups, while the condyle height was statistically significant, although all the condylar parameters were greater among non-TMD patients than the TMD patients. Regarding the intra-joint condylar positions, the anteroposterior (APJCP) and vertical (VJCP) condyle positions showed highly significant differences among groups in which the condylar position was more superior (3.28 ± 1.05 mm) in the TMD group in comparison with the non-TMD group (3.74 ± 0.93 mm) and more posterior in the TMD group (− 7.09 ± 20.84 mm) than in the non-TMD group (11.88 ± 17.75 mm).

In the measurements of joint spaces (Table 6), our findings revealed significant differences between the two groups. TMD group showed increased anterior (AJS) (2.73 ± 0.70 mm) and medial (MJS) (3.95 ± 1.08 mm) joint spaces relative to non-TMD patients (2.20 ± 0.73 and 2.73 ± 0.88 mm, respectively). In comparison, the superior (SJS) and posterior (PJS) joint spaces were reduced in the TMD group (3.74 ± 0.93 and 2.41 ± 0.82 mm) as compared to the non-TMD group (4.27 ± 1.37 and 2.84 ± 1.04 mm).

**Table 6:** Comparative statistical analysis of the temporomandibular joint spaces measurements between the TMD and non-TMD groups

Mandibular joint spaces measurements	Group				*P*-value
	TMD group		Non-MD group		
	Mean	SD	Mean	SD	
AJS	2.73	0.70	2.20	0.73	**0.000**
SJS	3.74	0.93	4.27	1.37	**0.005**
PJS	2.41	0.82	2.84	1.04	**0.004**
MJS	3.95	1.08	2.73	0.88	**0.000**

## Discussion

TMD is a common health issue, and it is an umbrella term that includes a variety of signs and symptoms influencing muscles of mastication, TMJ, and dentoalveolar components [22]. In this aspect, it is considered a musculoskeletal disorder causing orofacial pain of non-dental origin affecting the head, face, and related structures [23]. TMD is a multifactorial disease with numerous direct and indirect causal factors [24].

The present study investigated, in a 3D view, dimensional and positional osseous characteristics of TMJ structures in normodivergent facial patterns with and without temporomandibular disorders following an established method by Alhammadi et al. [20, 25].

Several studies [19, 26, 27] evaluated the association between the condyle and mandibular fossa in the hypodivergent and hyperdivergent skeletal patterns. On the other hand, other studies [15, 16, 28] evaluated TMJ features in patients with different forms of TMD: myalgia, disc displacement with reduction, and disc displacement without reduction. However, 3D dimensional and positional osseous characteristics of TMJ structures in normodivergent skeletal patterns have not been evaluated comprehensively in patients with or without TMDs. In this study, all participants have comparable skeletal patterns without anteroposterior or vertical discrepancies to ensure skeletal demographic standardization with minimal variations.

In the current study, the mandibular condyle inclination in horizontal and vertical planes revealed highly significant differences between both groups. This is partly similar to De Stefano et al. [29] study who indicated that mandibular condyle inclination might differ in TMJs with different disc positions, and they stated that a more medial horizontal condylar inclination and a more posterior sagittal condylar inclination were linked to TMDs like disc displacement without reduction. Also, Busato et al. [30] and Raustia et al. [31] considered that horizontal condyle inclinations were significantly different between subjects with normal joints and those having disc incoordination, whereas Amorin et al. [32] revealed no association between the horizontal inclination of the mandibular condyle and disc displacement. This finding infers that the change in the disc position in TMD patients is mainly by displacement in the anterior and medial direction leading to horizontal and vertical inclination changes as a result of the bone remodeling, respectively. This change is reflected in the remodeling of the glenoid fossa in the three dimensions, as shown in the current results too.

Our study revealed that the condyle in TMD patients was more superiorly, posteriorly, and laterally positioned in the glenoid fossa; this is shown in both aspects, the position relative to the fixed basal reference planes and within the joint measurements. This might indicate that the long-standing positioning of the disc in the anteromedial position pushes the condyle into posterior and lateral position, and the superior joint space that was occupied by the disc above the condyle head becomes less due to the same dynamic effect, so the condyle moved vertically to occupy this space. This is in agreement with Dalili et al. [33] who stated that the centric location of the condyle in the mandibular fossa was a common position. But, this disagreed with Alhammadi et al. [20] who reported that the condyles in non-TMD individuals were more positioned in a non-centric location in the glenoid fossa. Also, Imanimoghaddam et al. [34] and Incesu et al. [35] sated that the posterior condylar position was the most common position among TMD cases. These significant changes also reflected by the significant differences of the mandibular fossa position in the three planes of space between both groups; this might have occurred as a secondary change in the form of bone remodeling following the condylar positional changes in the three planes. The most significant condylar positional change was in the vertical direction, which was also demonstrated by the significant increase in the mandibular fossa height in the TMD group in comparison with the normal patients.

The current findings showed no statistically significant differences in the condyle width in both studied groups. Likewise, Imanimoghaddam et al. [34] reported a non-significant relationship between anterior disc displacement with reduction and alterations in condylar width too. On the contrary, Okur et al. [36] evaluated condylar width by CT, and a significant difference was revealed between normal and symptomatic cases. Also, Seo et al. [37] demonstrated that the condyle width was less in anterior disc displacement with reduction in comparison with asymptomatic patients.

Regarding the condylar length, our findings did not demonstrate any significant difference between normal subjects and TMD patients, similar to Imanimoghaddam et al. [34] results who reported a non-significant difference regarding condyle length between normal TMJs and patients with anterior disc displacement with reduction. However, these results are not consistent with the study conducted by Yasa and Akgül [28]; they revealed that the condylar length was smaller among anterior disc displacement with reduction patients than in asymptomatic patients.

In our study, condyle height was less among TMD cases compared with normal subjects; this is consistent with Mohamed et al. [38] who reported that condylar height was decreased in TMD group in comparison with normal subjects and disagreed with the finding of Seo et al. [37] who stated that condyle height did not show a significant difference between healthy joints and patients with anterior disc displacement with reduction. Mathematically, this change is considered as false positive due to the use of the local reference line in this measurement aided by a change in the vertical condylar position relative to this line rather than the actual change in the condylar length.

The superior and posterior joint spaces showed a significant reduction, while anterior and medial joint space increased among TMD patients. This was in agreement with Yasa and Akgül [28] who reported a significant difference in joint space measurements between normal and TMJ dysfunction cases but was inconsistent with Imanimoghaddam et al. [34] who conducted that superior and posterior joint spaces showed no significant differences between normal subjects and TMD patients (*P* = 0.36 and *P* = 0.7, respectively). This is another indication that the changes in the disc position affect the whole TMJ system. In this case, the reduction in superior and posterior joint spaces is another indication of the condyle’s superior and anterior reactive positioning, respectively. At the same time, the increased anterior and medial joint spaces reflect the posterior and lateral change in the condyle position, as evident elsewhere.

One of the limitations of this study is that it is limited to adult patients, and including growing patients may change the finding of this study; another limitation is that it is limited to specific ethnic groups, and the finding cannot be generalized to other ethnicities or populations. The assessment was limited to the osseous structures; the use of MRI to examine the soft tissue component is recommended in similar future studies.

## Conclusion

The findings of this study revealed a significant association between TMDs and TMJ positional and morphological osseous characteristics; the patients diagnosed with TMD showed significantly different mandibular fossa positions in all planes, fossa height, condylar positions, and the horizontal and vertical condylar inclinations. The AJS and MJS increased while the SJS and PJS reduced in TMDs patients.

## Supplementary information

Below is the link to the electronic supplementary material.

Supplementary file1 (DOCX 5583 KB)

Supplementary file2 (DOCX 7787 KB)
