# Supplementary material for: Dimensional and positional temporomandibular joint osseous characteristics in normodivergent facial patterns with and without temporomandibular disorders
Source: Clin Oral Investig. 2023 Jun 26;27(9):5011–20. doi: 10.1007/s00784-023-05120-0 (PMC10492742; doi:10.1007/s00784-023-05120-0)
Supplement: Supplementary file 1 — Supplementary file1 (DOCX 5583 KB) [file 784_2023_5120_MOESM1_ESM.docx]

| **No.** | **Landmark** | **Definition** |
| --- | --- | --- |
| **Skeletal Landmarks (Fig. 1)** | | |
| **1** | **S** | The center point of the pituitary fossa in the middle cranial fossa in sagittal and axial views |
| **2** | **N** | The most anterior and midpoint of the fronto-nasal suture |
| **3** | **Or** | The most inferior and middle point of each infra-orbital rim |
| 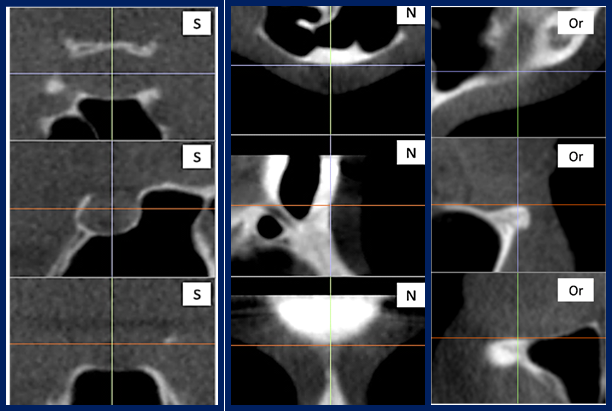 | | |
| **4** | **Po** | The most outer and superior bony points of the external acoustic meatus |
| **5** | **ANS** | The most anterior midpoint of the anterior nasal spine of the maxilla |
| **6** | **A point** | The deepest midpoint of the maxillary anterior surface |
| 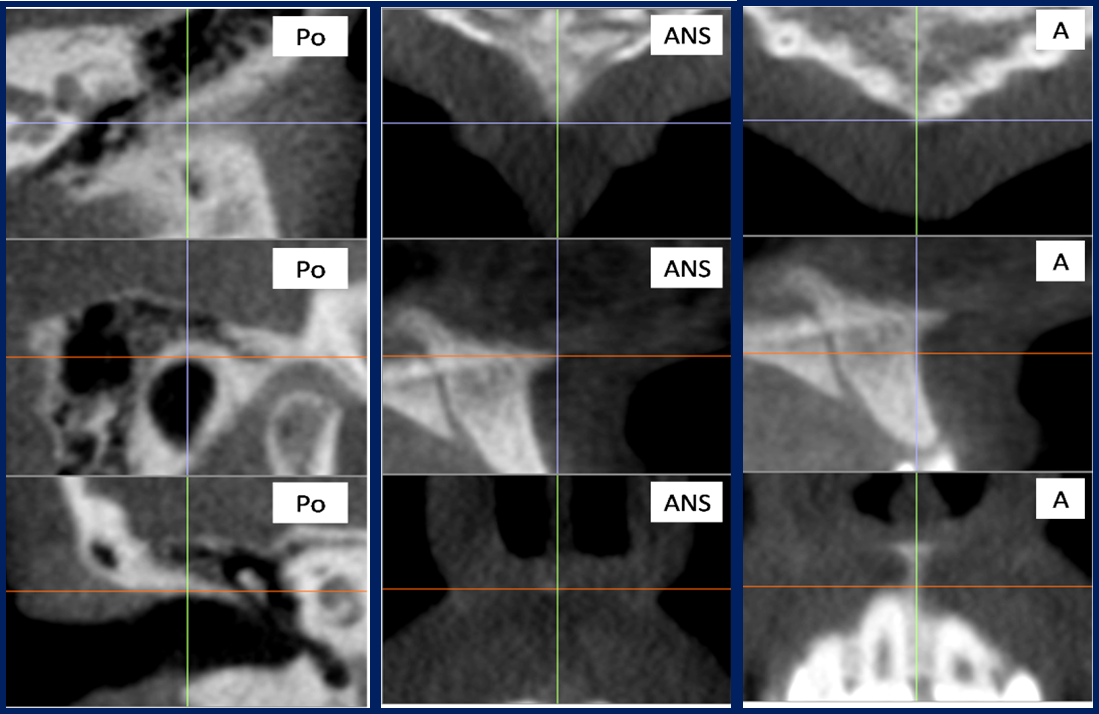 | | |
| **7** | **B point** | The deepest midpoint of the mandibular anterior surface |
| **8** | **Me** | The most inferior midpoint of the chin on the outline of the mandibular symphysis |
| **9** | **Go** | The right and the left midpoint on the angles of the mandible, halfway between the corpus and ramus |
| 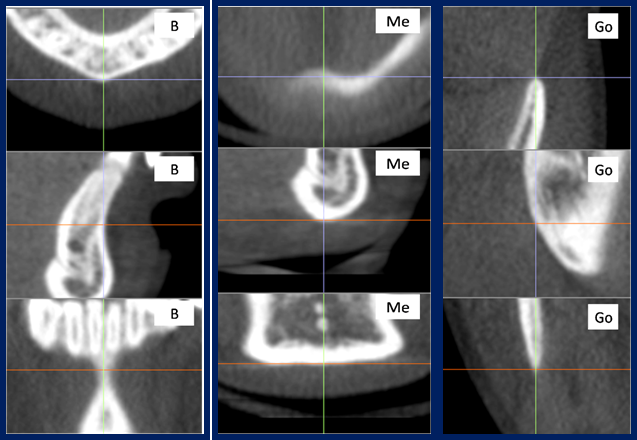 | | |
| **Temporomandibular Landmarks (Fig. 2)** | | |
| **1** | **MF** | The most superior and midpoint of the hard tissue right or left mandibular fossa region. |
| **2** | **AT** | The most inferior point of articular tubercle |
| **3** | **IM** | The most inferior point of internal auditory meatus |
| 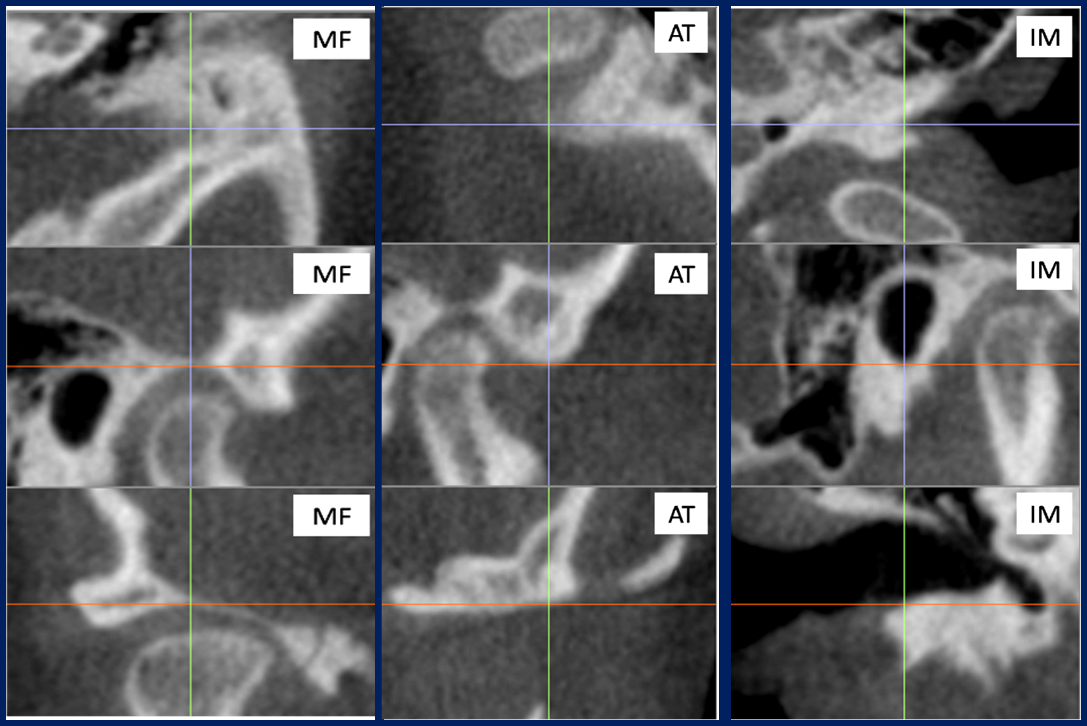 | | |
| **4** | **AFPi** | The most anterior and inferior point in the right or left anterior wall of the mandibular fossa |
| **5** | **AFPs** | The most superior point in the right or left anterior wall of the mandibular fossa |
| **6** | **PFPi** | The most posterior and inferior point in the right or left anterior wall of the mandibular fossa |
| **7** | **PFPs** | The most superior point in the right or left posterior wall of the mandibular fossa |
| 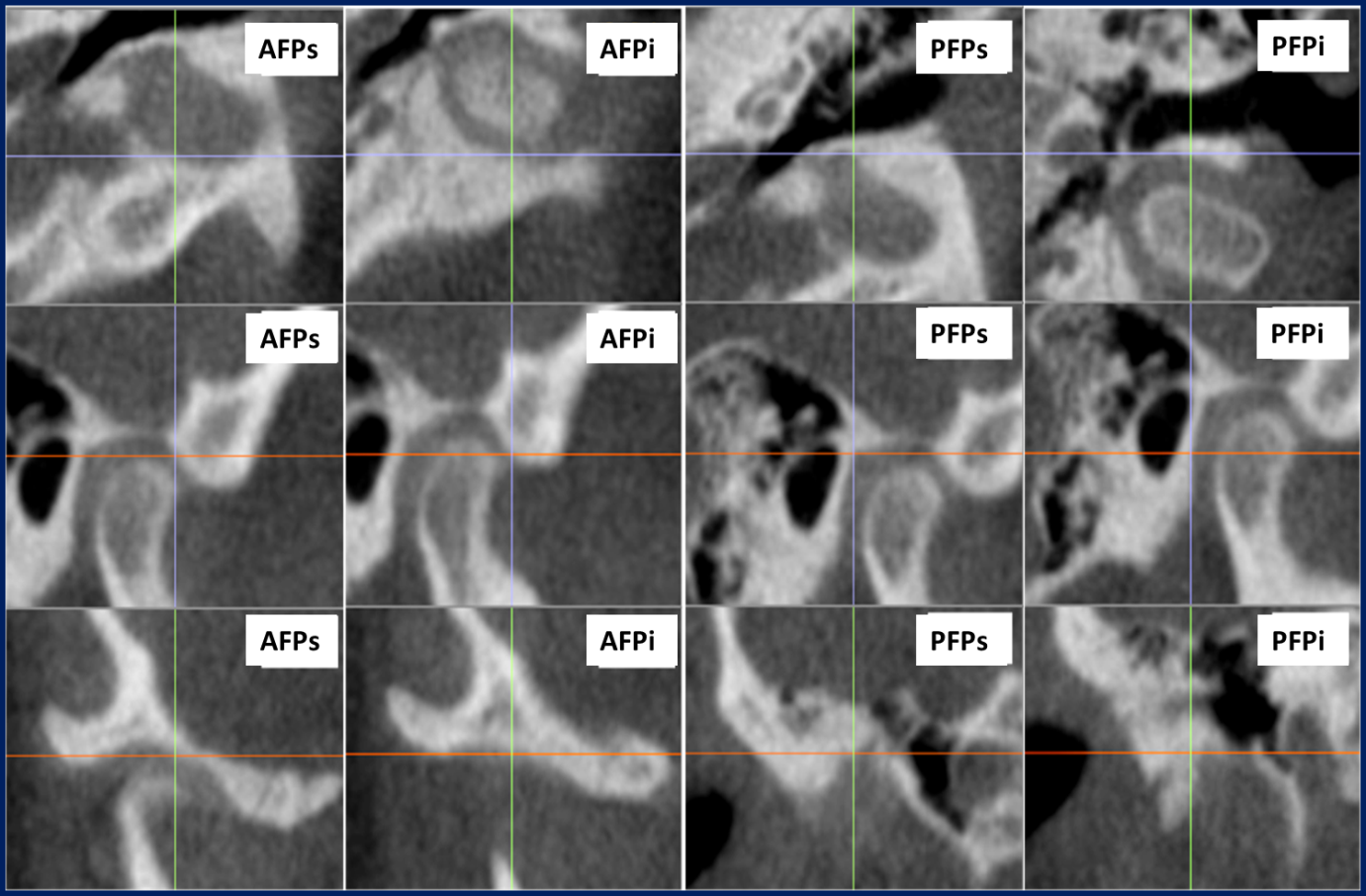 | | |
| **8** | **SCP** | The most right or left superior point of the condylar head |
| **9** | **LCP** | The most right or left lateral point of the condylar head |
| **10** | **MCP** | The most right or left medial point of the condylar head |
| **11** | **ACP** | The most right or left anterior point of the condylar head |
| **12** | **PCP** | The most right or left posterior point of the condylar head |
| 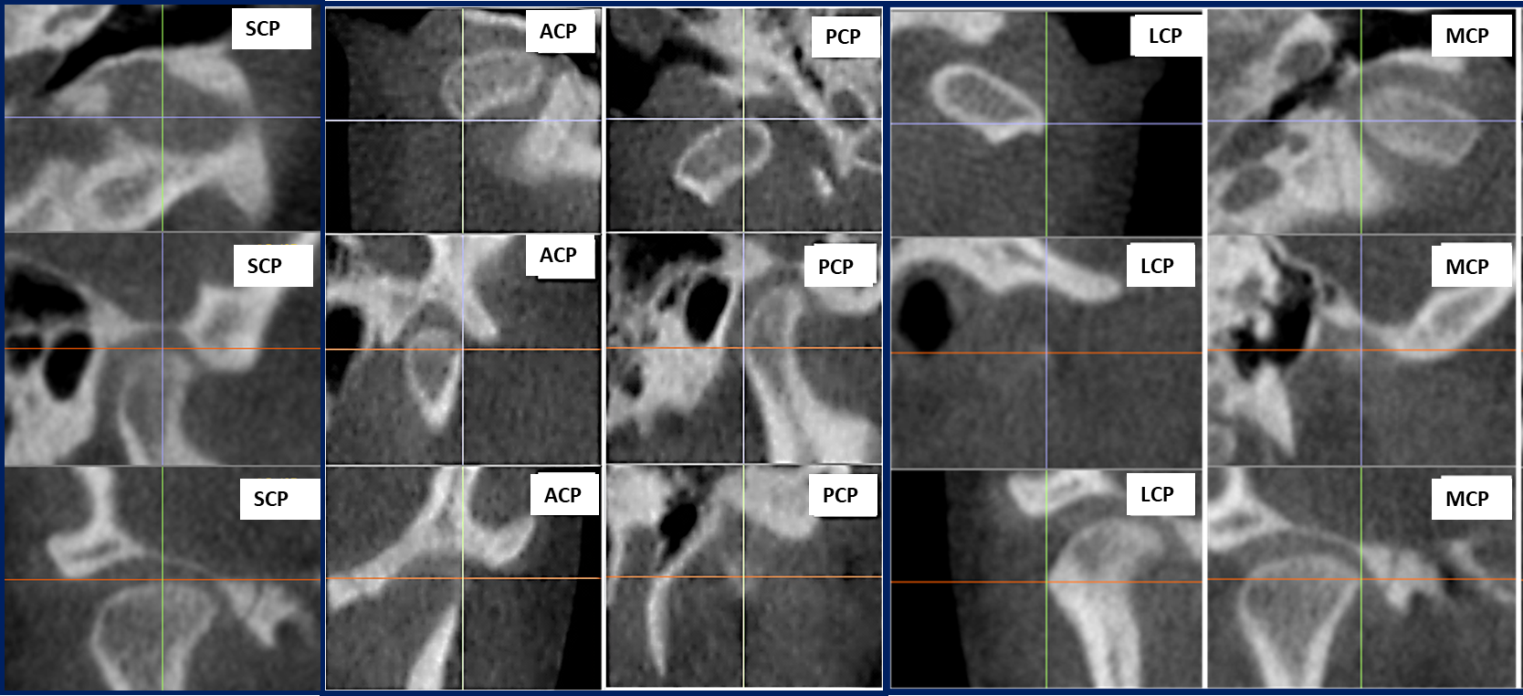 | | |
| **13** | **MJSF** | The most right or left lateral point of the medial wall of mandibular fossa. |
| **14** | **AJSF** | The most posterior point of the right or left anterior wall of the mandibular fossa opposed to the shortest anterior condylar-fossa distance |
| **15** | **AJSC** | The most anterior point of the right or left condyle opposed to the shortest anterior condylar-fossa distance |
| **16** | **PJSF** | The most anterior point of the right or left posterior wall of the mandibular fossa opposed to the shortest posterior condylar-fossa distance |
| **17** | **PJSC** | The most posterior point of the right or left condyle opposed to the shortest posterior condylar-fossa distance |
| 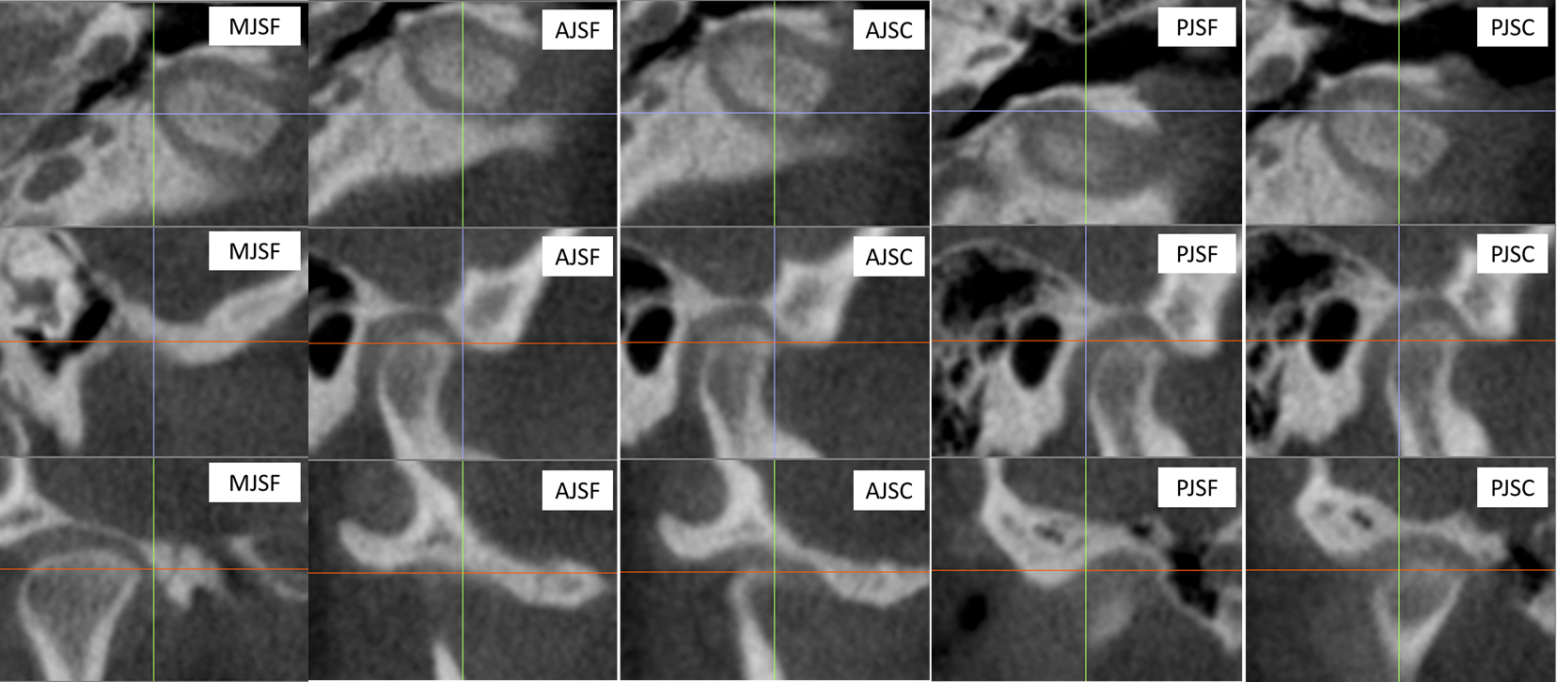 | | |
