# Supplementary material for: Dimensional and positional temporomandibular joint osseous characteristics in normodivergent facial patterns with and without temporomandibular disorders
Source: Clin Oral Investig. 2023 Jun 26;27(9):5011–20. doi: 10.1007/s00784-023-05120-0 (PMC10492742; doi:10.1007/s00784-023-05120-0)

I- Three dimensional skeletal anteroposterior and vertical measurements

| 1 | ANB | Skeletal anteroposterior Jaw relation |
| --- | --- | --- |
| 2 | MP/SN | Skeletal vertical Jaw relation |


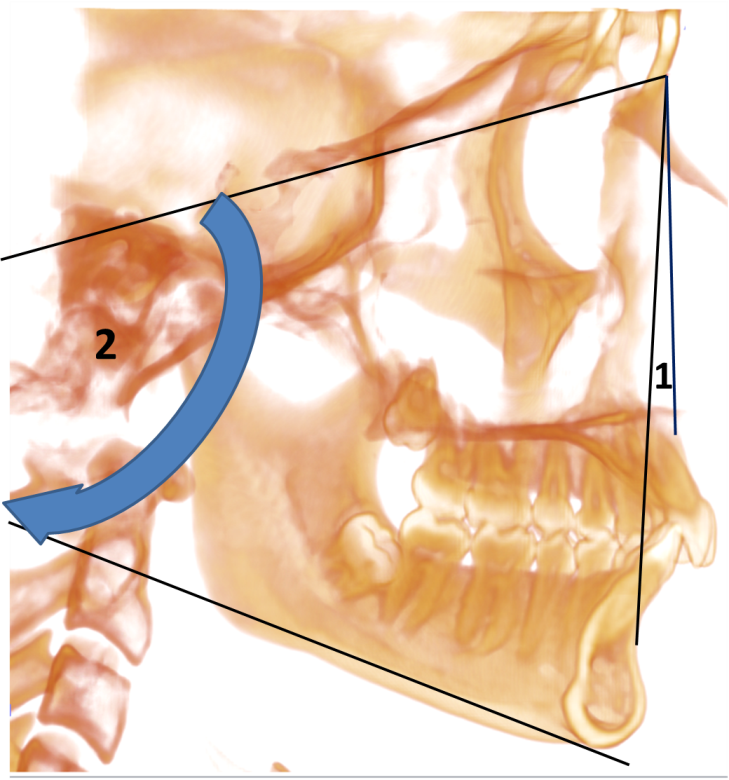


II- Three dimensional mandibular fossa measurements

| 3 | MFPVP | Mandibular fossa vertical position |
| --- | --- | --- |
| 4 | MFPAP | Mandibular fossa anteroposterior position |
| 5 | MFPML | Mandibular fossa mediolateral position |


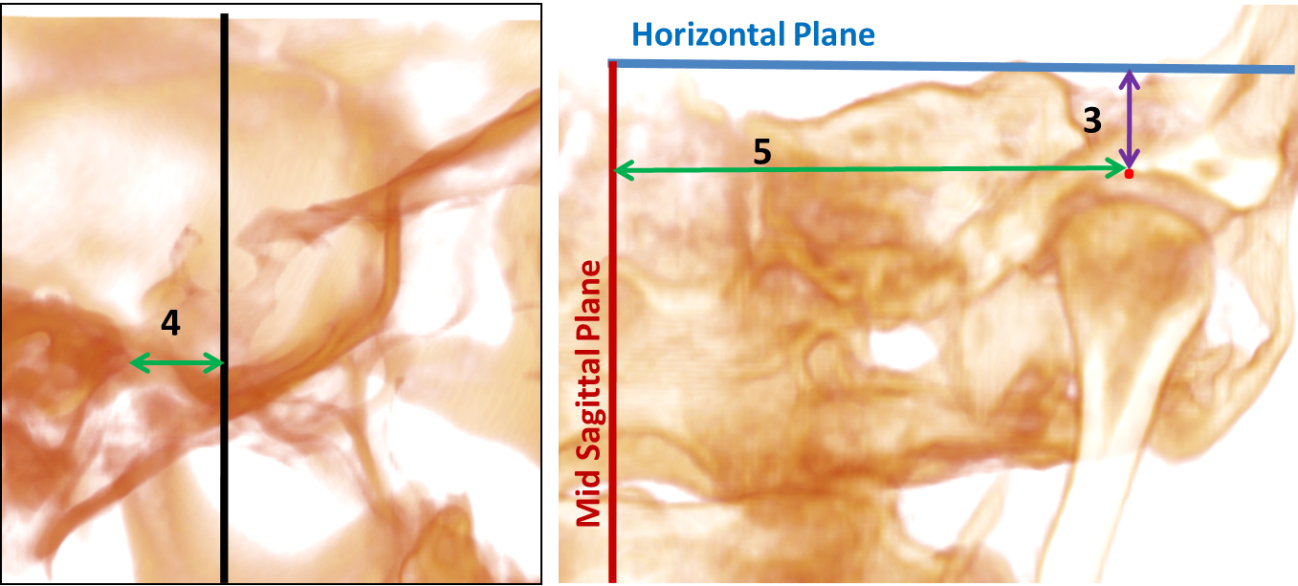


| 6 | GFH | Mandibular fossa height |
| --- | --- | --- |
| 7 | GFW | Mandibular fossa width |
| 8 | AFLHP | Mandibular fossa anterior wall inclination |
| 9 | PFLHP | Mandibular fossa posterior wall inclination |


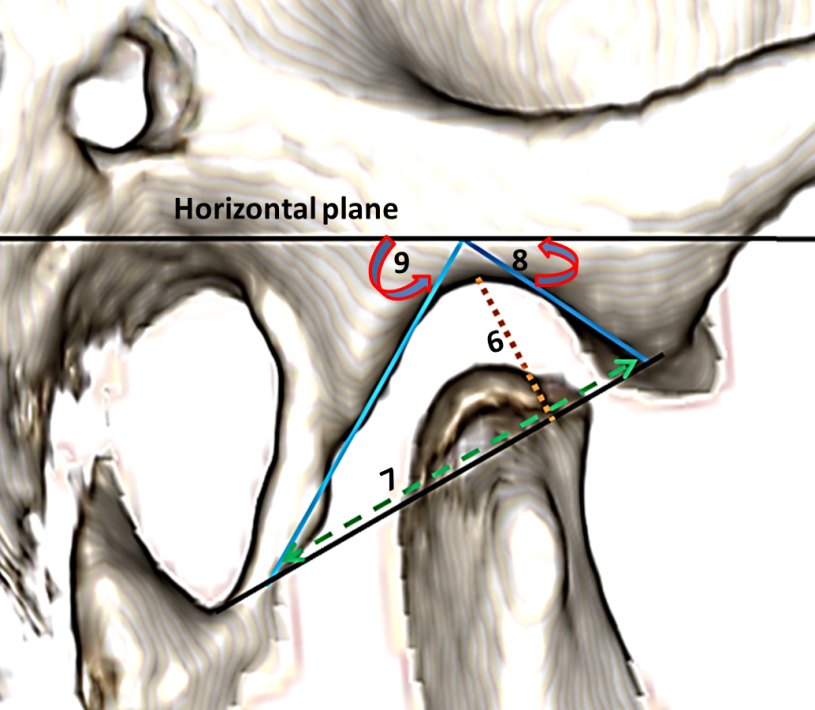


III- Three dimensional mandibular condyle measurements

| 10 | HCI | Mandibular condyle horizontal inclination |
| --- | --- | --- |
| 11 | VCI | Mandibular condyle vertical inclination |
| 12 | APCI | Mandibular condyle anteroposterior inclination |


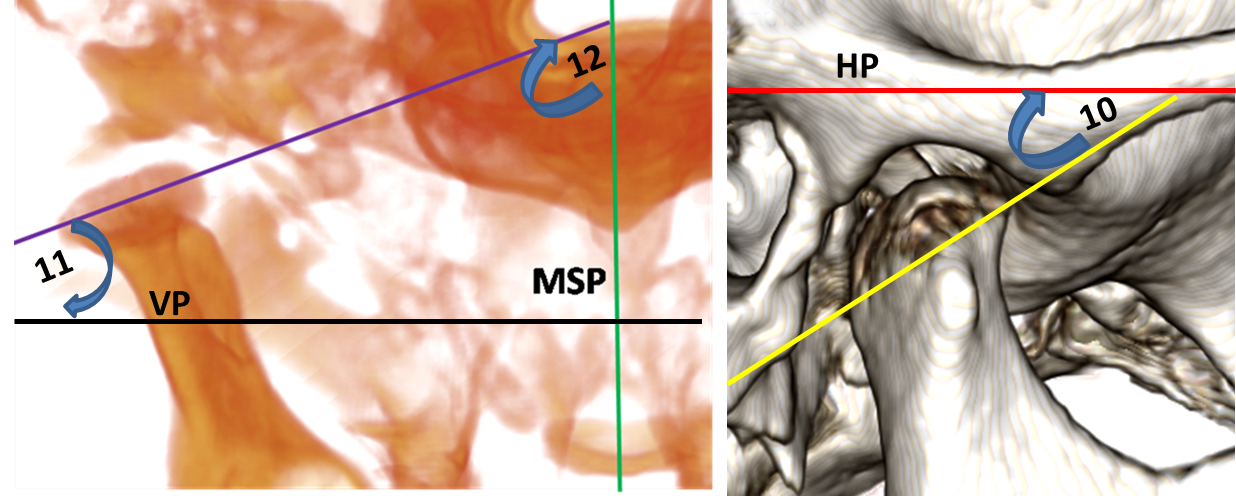


| 13 | VCP | Mandibular condyle vertical position |
| --- | --- | --- |
| 14 | APCP | Mandibular condyle anteroposterior position |
| 15 | MLCP | Mandibular condyle mediolateral position |


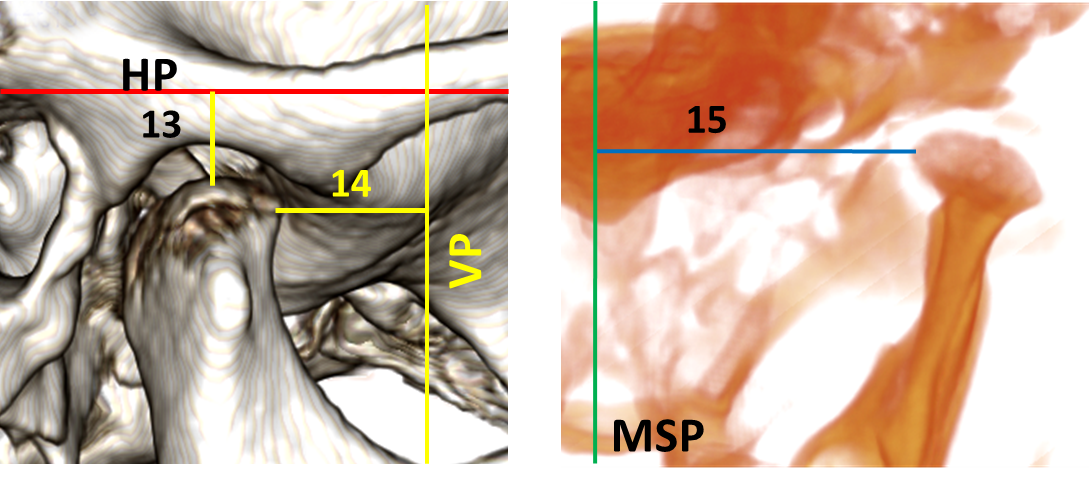


| 16 | CL | Condylar length |
| --- | --- | --- |
| 17 | CW | Condylar width |
| 18 | CH | Condylar height |
| 19 | VCJP | Based on calculation of other measurements (refer to table 2) |
| 20 | APCJP | Based on calculation of other measurements (refer to table 2) |


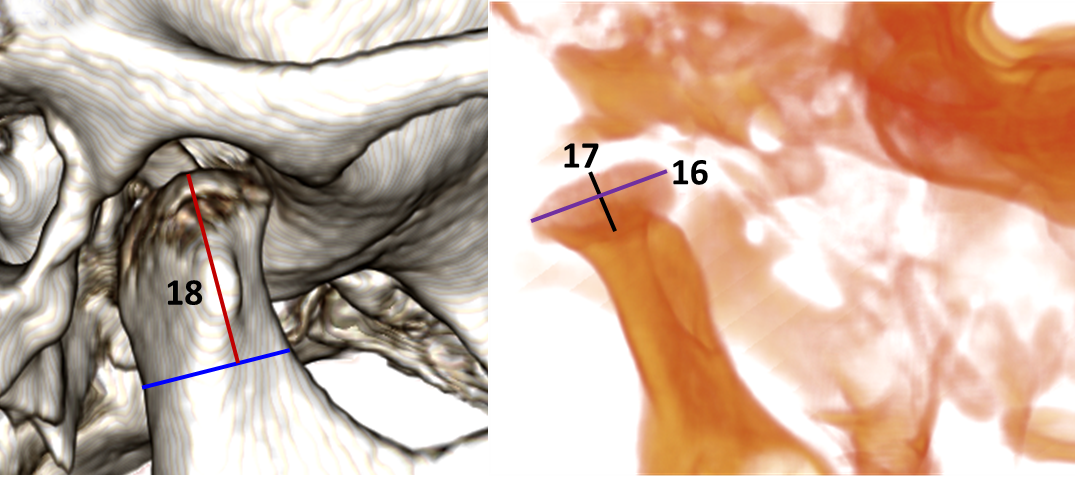


IV- Three dimensional temporomandibular joint spaces measurements

| 21 | AJS | Anterior Joint Space |
| --- | --- | --- |
| 22 | PJS | Posterior joint space |
| 23 | SJS | Superior joint space |
| 24 | MJS | Medial joint space |


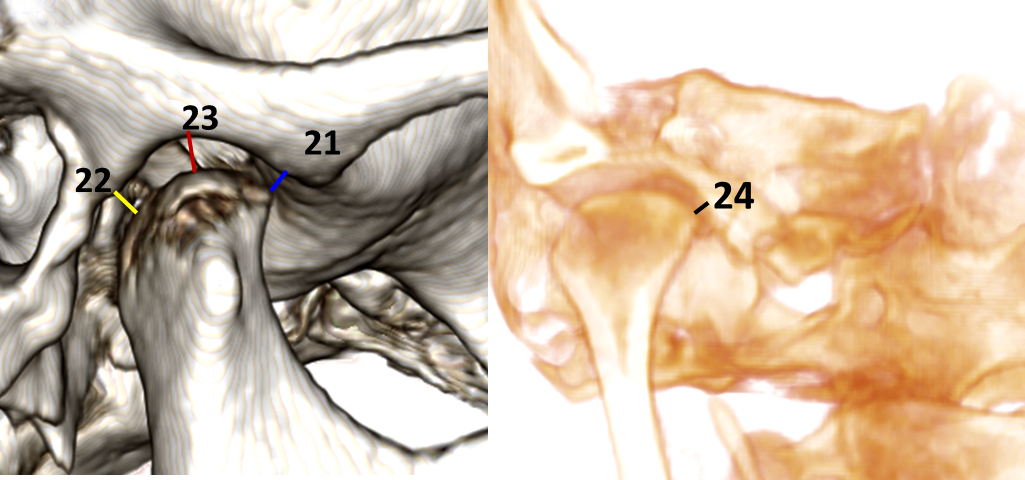

Supplement: Supplementary file 2 — Supplementary file2 (DOCX 7787 KB) [file 784_2023_5120_MOESM2_ESM.docx]
